# Induction of multiple myeloma cancer stem cell apoptosis using conjugated anti-ABCG2 antibody with epirubicin-loaded microbubbles

**DOI:** 10.1186/s13287-018-0885-2

**Published:** 2018-05-21

**Authors:** Fangfang Shi, Miao Li, Jing Wang, Di Wu, Meng Pan, Mei Guo, Jun Dou

**Affiliations:** 1 Department of Oncology, Zhongda Hospital, School of Medicine, Southeast University, Nanjing, 210009 China; 2 Department of Pathogenic Biology and Immunology, School of Medicine, Southeast University, 87# Ding Jiaqiao Rd., Nanjing, 210009 China; 3 Department of Gynecology & Obstetrics, Zhongda Hospital, School of Medicine, Southeast University, Nanjing, 210009 China

**Keywords:** Multiple myeloma, Cancer stem cells, Epirubicin, Microbubble, Ultrasound

## Abstract

**Background:**

Multiple myeloma (MM) currently remains largely incurable. Cancer stem cells (CSCs) are believed to be responsible for drug resistance and eventual relapse. In this study, we exploited a novel agent to evaluate its inhibitory effect on MM CSCs.

**Methods:**

Epirubicin (EPI)-loaded lipid microbubbles (MBs) conjugated with anti-ABCG2 monoclonal antibody (EPI-MBs + mAb) were developed and their effect on MM 138^−^CD34^−^ CSCs isolated from human MM RPMI 8226 cell line plus ultrasound exposure in vitro and in vivo in a nonobese diabetic/severe combined immunodeficient mouse model were assessed.

**Results:**

EPI-MBs + mAb combined with ultrasound led to a significant decrease in the clone formation ability and the mitochondrial membrane potential along with an increase in MM CSC apoptosis. Moreover, treatment with EPI-MBs + mAb with ultrasound exposure remarkably inhibited the growth of MM CSC-derived tumors in xenograft nonobese diabetic/severe combined immunodeficient mice compared with a single agent or EPI-MBs + mAb without ultrasound exposure. The inhibitive efficacy was also correlated with an increased expression of caspase-3, Bax, and TUNEL and decreased expressions of PCNA, Bcl-2, and CD31.

**Conclusions:**

Our findings reveal that the EPI-MBs + mAb combined with therapeutic ultrasound may confer an effective approach for treatment of MM by induction of an apoptotic pathway in MM CSCs.

## Background

Multiple myeloma (MM) is a plasma cell malignancy characterized by the growth of MM cells in the bone marrow, and it is still incurable. Although hematopoietic stem cell transplantation and advances in chemotherapy, such as proteasome inhibitors and immunomodulatory drugs, have significantly achieved lasting remission and increased survival rates in patients with MM, most patients eventually relapse [1–3]. Cancer stem cells (CSCs), which show the features of self-renewal, multidirectional differentiation, and multidrug resistance, are thought to be the major cause of tumor chemoresistance and recurrence. MM treatment failure has demonstrated that current therapeutic agents are not enough to eradicate MM CSCs [4–7].

Epirubicin (EPI) is one of the anthracycline drugs commonly used for therapy in MM, breast, sarcoma, and many other malignant tumors. Since EPI can be distributed both in tumor and normal tissue when injected intravenously it shows various adverse reactions, including digestive tract toxicity, myelosuppression, functional lesions in the liver and kidney, and so on [8, 9]. To reduce the side effects and enhance the local drug concentration in MM chemotherapy, we hypothesize that it may be feasible to use an ultrasound-targeted microbubble destruction (UTMD) technique combined with a specific antibody as a drug targeting MM CSCs to increase anti-MM efficacy [10].

Microbubbles (MBs) are a rapid-development drug-delivery system with many advantages, including high security and drug-loading capability using the different prepared methods. The biological effect of UTMD on MBs might enhance the capability of MB drug delivery. For example, EPI was released from the targeting MBs when ultrasound exposure was applied to tumors, which enhanced the EPI concentration in local tumor tissue and reduced the cytotoxicity of EPI to the host body. Therefore, drug-loaded MBs conjugated with antibody in combination with UTMD appears to be a promising strategy for the treatment of tumors [11–13].

The high drug-efflux capability of MM CSCs is likely to be a major cause of drug resistance in MM. ABCG2 is one of the ATP-binding cassette (ABC) transporters that represents the family of transmembrane proteins. It is known that ABCG2 is highly expressed in MM CSCs, showing a strong activity to efflux cytotoxic compounds [14–17]. We previously demonstrated that the combination of anti-ABCG2 monoclonal antibody (mAb) and paclitaxel iron oxide nanoparticles resulted in improving anti-MM CSC efficacy. In the present study, to reduce both drug resistance and the adverse reaction to EPI, we exploited a novel agent using EPI-loaded lipid MBs conjugated with anti-ABCG2 mAb to assess its effect on MM 138^−^CD34^−^ CSCs under ultrasound exposure.

Here, we show that the combination of EPI-MBs + mAb with therapeutic ultrasound could achieve a significant inhibition of MM CD138^−^CD34^−^ CSC clone formation and induction of CSC apoptosis in vitro, as well as a reduction in MM CD138^−^CD34^−^ CSC-derived tumor growth in nonobese diabetic/severe combined immunodeficient (NOD/SCID) mice compared with using EPI alone or EPI-MBs + mAb without ultrasound exposure. This synergy of the combination of EPI-MBs + mAb with UTMD may be considered for further preclinical trials in the treatment of drug-resistant MM.

## Methods

### Cell Line and Mice

Human MM RPMI 8226 cells were purchased from the Cell Bank of the Chinese Academy of Sciences (Beijing, China). Cells were cultured in complete medium consisting of RPMI 1640, 2 mM l-glutamine, 100 U/mL penicillin, 100 μg/mL streptomycin, and 10% fetal bovine serum at 37 °C in a humidified incubator containing 5% CO_2_. NOD/SCID mice at 6 weeks of age (17–18 g weight) were purchased from Beijing Weitong Lihua Experimental Animal Technology Co., Ltd., China. Mice were maintained in a pathogen-free facility that has a 12-h light/dark cycle and relative humidity ranged from 40% to 50% at 24 °C. All the animal experiments were performed in compliance with the Guidelines of the Animal Research Ethics Board of Southeast University. This ethics board also approved the animal studies.

### Preparation of EPI-MBs + mAb

The EPI-MBs + mAb were prepared as described in our previously published paper [18]. CY7.5 labeled EPI-MBs and EPI-MBs + mAb were obtained by adding proper CY7.5 (Lumiprobe, LLC) to chloroform in the preparation [19, 20].

### Isolation of MM CD138^−^CD34^−^ CSCs

CD138^−^CD34^−^ cells were isolated from the human MM RPMI 8226 cell line by a magnetic activated cell sorting method (Miltenyi Biotec, Gladbach, Germany) following our previous study protocol [16, 18, 21]. We named CD138^−^CD34^−^ cells as MM CSCs.

### EPI uptake efficiency of MM CD138^−^CD34^−^ CSCs

MM CD138^−^CD34^−^ CSCs (1 × 10^6^) in a vial were respectively incubated with phosphate-buffered saline (PBS), EPI, or EPI-MBs + mAb for 30 min. The EPI concentration was 10 μg/mL in the vial, and the vial was placed upside down to maximize the cell-MB interaction. Ultrasound exposure was performed for 40 s on vials at 0.5 W/cm^2^ [22]. The vial was cultured at 37 °C in an incubator containing 5% CO_2_ for an additional 30 min. MM CD138^−^CD34^−^ CSCs in the vial were washed three times with PBS to remove MBs + mAb and excess EPI (1000 rpm/8 min), and stained with 4’,6-diamidino-2-phenylindole (DAPI) for 10 mins, and then washed with PBS three times (2000 rpm/5 min). The fluorescence intensity of coal maceral in MM CD138^−^CD34^−^ CSCs was observed under a confocal fluorescence microscope [23]. The process was performed in the dark at room temperature.

### Colony formation assay

MM CD138^−^CD34^−^ CSCs (1 × 10^6^) were treated with different agents following our previous protocol [18]. One hundred single-cell suspension cells were resuspended in 0.8 mL conditioned medium (CM) containing 0.3% low melting temperature agarose (Promega, USA) and were plated in triplicate on 24-well plates over a base layer of 0.8 mL CM containing 0.6% low melting temperature agarose. The plates were incubated for 10–14 days until colonies were formed. Colony diameters larger than 75 μm or colonies containing more than 50 cells were then counted as one positive colony according to previous reports [4, 24].

### Analysis of mitochondrial membrane potential and cell cycle

For the mitochondrial membrane potential, 1 × 10^6^ MM CSCs treated with the different agents were washed twice with PBS and then resuspended in 500 μL JC-1 buffer for 15–20 min in incubators at 37 °C with 5% CO_2_. Cells were washed in 1 × 500 μL incubation buffer twice (2000 rpm/5 min) and were resuspended in incubation buffer and analyzed by flow cytometry (FCM) [18, 25].

For the cell cycle analysis, 1 × 10^6^ MM CSCs treated with the different agents were fixed overnight with 70% (w/v) ice-cold ethanol. Cells were resuspended in 1 mL PBS containing 40 μg/mL Annexin V/propidium iodide (PI) and 500 U/mL RNase A. Following incubation for 30 min in the dark at room temperature, cells were analyzed by FCM using the system modfit software. The PI fluorescence signal peak versus the integral was used to discriminate G2-M cells from G0-G1 doublets [26]. The cell cycle and JC-1 apoptosis detection kits were obtained from Keygen Biotech (China).

### Detection of EPI-MBs + mAb binding to MM tissues and ultraphonic echo intensity

MM CD138^–^CD34^–^ CSCs (1 × 10^6^; 100 μL) were mixed with matrigel (BD Biosciences; 100 μL in volume) and injected subcutaneously (s.c.) into the right dorsal side of NOD/SCID mice. When the grafted s.c. tumor volume reached 0.8–1.0 cm, the mice were divided into EPI-MBs + mAb (Anti-ABCG2 mAb; Cell Signaling Corporation) and EPI-MBs groups (three per group). In the EPI-MBs + mAb group, 0.2 mL EPI-MBs + mAb (MBs 3 × 10^9^/mL, EPI 0.5 mg/mL) was injected into the mice through the tail vein. The same amount of EPI-MBs were injected accordingly in the other groups. Ultrasound imaging of subcutaneous tumors was performed before the injection of EPI-MBs + mAb and 30 min after the injection with a color Doppler diagnostic apparatus (My Lab Twice) equipped with a 5–8 MHz broadband linear transducer (mechanical index 0.06). Ultrasound issued by the same transducer (mechanical index 0.4) was then performed for 3 min on tumors [27]. Thirty minutes later, two mice in each group were executed, and the subcutaneous tumors were removed for frozen sectioning. The distribution of EPI-MBs + mAb and EPI-MBs in the MM tissues was observed under a light microscope.

### Treatment of MM-bearing mice

The human RPMI 8226 MM CSC xenograft NOD/SCID mice were randomly divided into PBS, EPI, EPI-MBs + mAb, and EPI-MBs + mAb + ultrasound (US) groups (six per group). Then, 200 μL of different agents including PBS, EPI (5 μg/kg), and EPI-MBs + mAb (EPI 5 μg/kg + mAb 0.5 μg/g) were s.c. injected into the mice after MM-bearing mice had been established for a total of six times and once every 3 days [28]. In the EPI-MBs + mAb + US group, UTMD was applied 30 min after the injection on tumors with a mechanical index of 0.4 for 3 min [22]. The tumor growth in NOD/SCID mice was monitored once 3 days for tumor volume by measuring two perpendicular tumor diameters from each mouse using calipers. The survival time was also observed in the remaining mice.

### Western blot

Tumor tissues were collected from each mouse and homogenized for detection of P-p65 and P-IκBα expression [29]. According to the manufacturer’s protocol, the proteins were separated using sodium dodecyl sulfate-polyacrylamide gel electrophoresis. The membrane was blocked with the buffer containing 10% fat-free dry milk; the rabbit antihuman P-p65, P-IκBα, and β-actin antibodies were respectively used as the primary antibody for 2 h, and the membrane was rinsed for 5 min with an antibody wash solution three times before adding a secondary horseradish peroxidase-conjugated anti-rabbit antibody for 1 h at room temperature. The following steps were performed according to Kit’s protocol [18].

### Immunohistochemistry and histopathology of tumor tissues

Immunostaining was performed as previously reported [30]. Briefly, 5-μm thin formalin-fixed and paraffin-embedded tumor sections were incubated with the rabbit anti-mouse/human caspase-3, Bax, TUNEL, PCNA (proliferating cell nuclear antigen), Bcl-2, and CD31, respectively, overnight at 4 °C. The antibody concentration was 1:600. The samples were then labeled with horseradish peroxidase-conjugated streptavidin (Invitrogen) and the chromogenic reaction was developed using the Liquid DAB Substrate Pack according to the manufacturer’s instructions. The stained cells from random and nonoverlapping fields were counted under a magnification of ×100 or ×400. Meanwhile, the sections were stained with hematoxylin and eosin for microscopic examination. All sections were observed in a blinded fashion and were photographed using a 400× normal light microscope.

### Statistical analysis

The data were plotted as mean ± SD and analyzed for statistical significance by two-tailed paired Student’s *t* test or repeated measures analysis of variance (ANOVA). *P* values less than 0.05 were considered statistically significant. Analyses were performed with the SPSS 19.0 software package.

## Results

### Analysis of MM CD138^−^CD34^−^ CSCs uptake of EPI

**Fig. 1:**
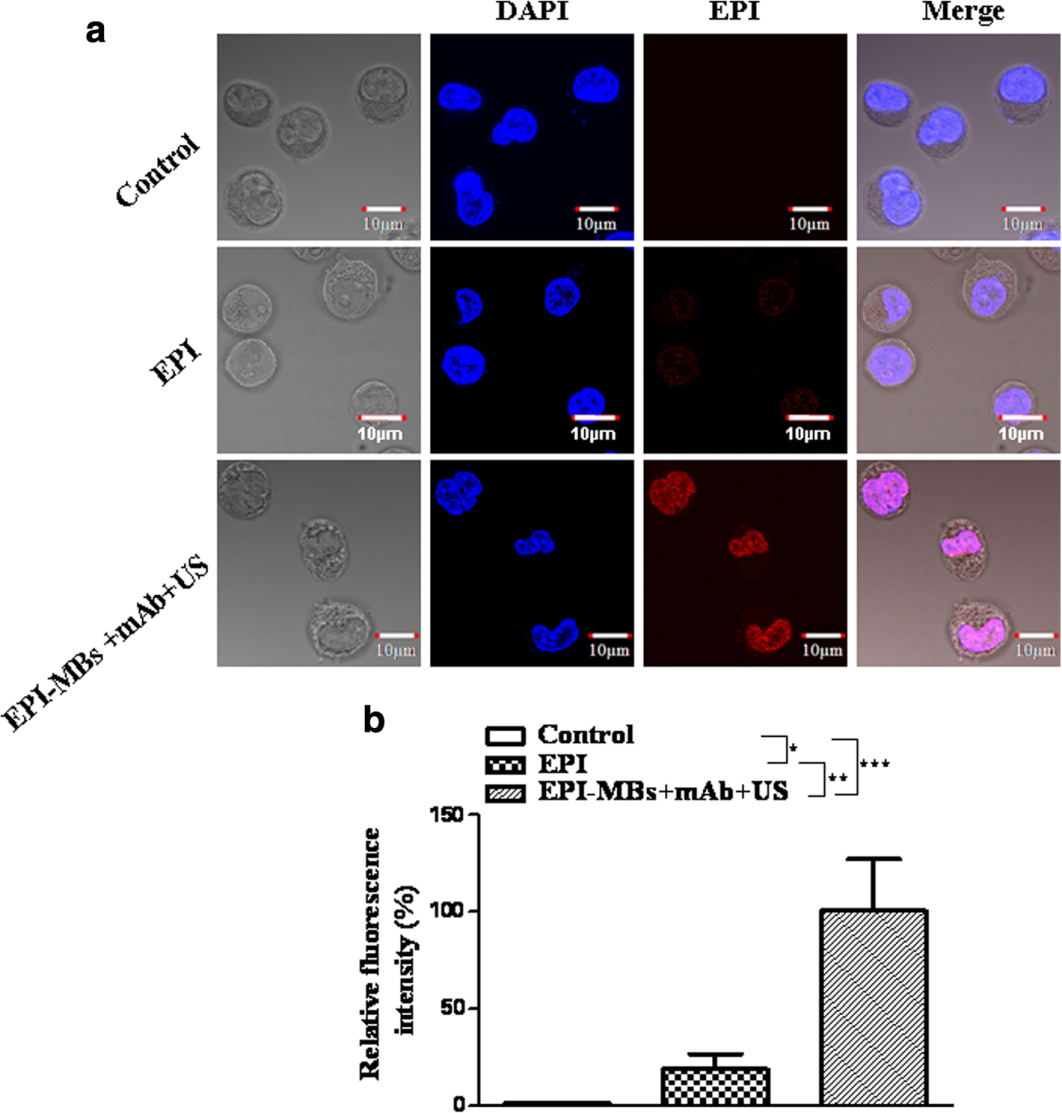
Analysis of epirubicin (EPI) entering MM CSCs. The images acquired from the confocal fluorescence microscopy were analyzed with Image J software, and the fluorescence intensity of cells in EPI-MBs + mAb + US was set to 100 to provide a basis for comparison. The relative fluorescence intensity of various groups was calculated. **a** Representative images show EPI entering MM CD138^−^CD34^−^ CSCs (red) 30 min after cells were respectively incubated with PBS (control), EPI (10 μg/mL), and EPI-MBs + mAb + US (0.5 W/cm^2^) and then stained with 4’,6-diamidino-2-phenylindole (DAPI) for 10 min as described in the Methods. Blue, red, and pink fluorescence intensity represents the DAPI (cellular nucleus), EPI (entering MM CSCs), and these merged, respectively. **b** Quantification of red fluorescence intensity in the different treated cells. *P < 0.05, ** *P* < 0.01, *** *P* < 0.005. EPI, epirubicin; mAb, monoclonal antibody; MB, microbubble; US, ultrasound

EPI-loaded MBs with conjugated anti-ABCG2 antibody (EPI-MBs + mAb) were prepared as described in our previous work [18]. To show the EPI uptake efficiency of MM CD138^−^CD34^−^ CSCs, we detected the fluorescence intensity in MM CD138^−^CD34^−^ CSCs by a confocal fluorescence microscopy. Figure 1a shows that MM CD138^−^CD34^−^ CSCs showed the highest fluorescence intensity among the three tested groups when CSCs were incubated with EPI-MBs + mAb combined with UTMD, indicating that more EPI (shown in red in the figure) accumulated in MM CD138^−^CD34^−^ CSCs, which was statistically significant compared with the EPI group (*P* < 0.01) or PBS (control) group (*P* < 0.001). Although EPI was partly taken up by MM CD138^−^CD34^−^ CSCs, the efficiency of EPI uptake was significant lower with no MBs + mAb and ultrasound exposure than that of EPI-MBs + mAb combined with ultrasound exposure, as shown in Fig. 1b. The results suggested that the EPI-MBs + mAb combined with UTMD could effectively target MM CD138^−^CD34^−^ CSCs and enhance EPI accumulation in MM CSCs in vitro.

### Effect of EPI-MBs + mAb combined with UTMD on MM CSCs

**Fig. 2:**
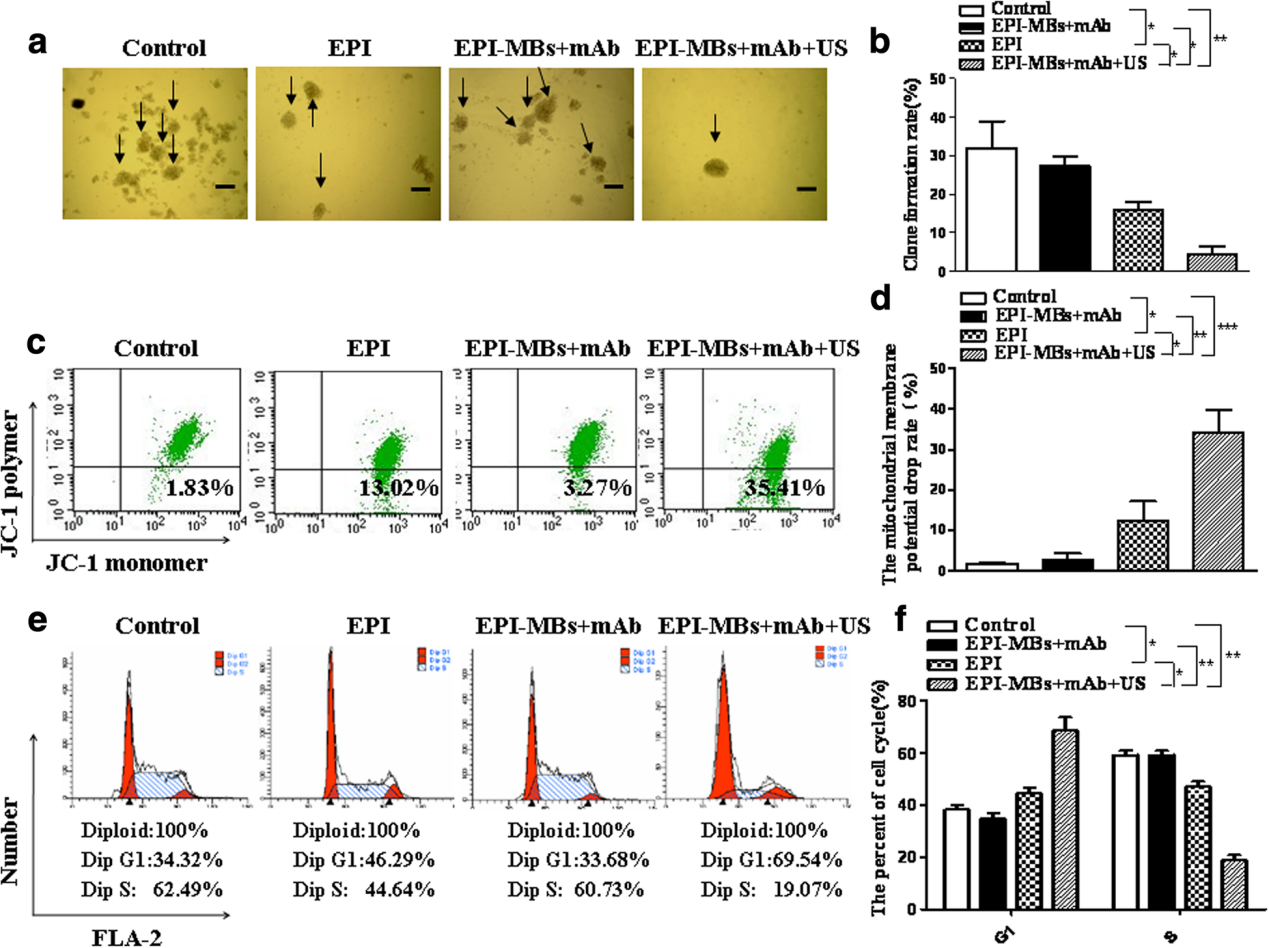
Analysis of clone formation, membrane potential, and cell cycle of MM CSCs. As described in the Methods, 1 × 10^6^ MM CSCs treated with various agents for 30 min were used for assay clone formation, membrane potential, and cell cycle analysis. **a** Images showing clone formation rate. **c,e** Changes in mitochondrial membrane potential and cell cycle were analyzed by FCM. **b,d,f** Statistical analysis of the clone formation rate and changes in mitochondrial membrane potential and cell cycle. *P < 0.05, **P < 0.01, ***P < 0.005. EPI, epirubicin; mAb, monoclonal antibody; MB, microbubble; US, ultrasound

First, we observed the effect of EPI-MBs + mAb combined with UTMD on MM CSCs in vitro. Figure 2a shows that the combined EPI-MBs + mAb with UTMD inhibited the clonogenic capability of MM CSCs in soft agar media. The clone formation rate was significantly lower in the EPI-MBs + mAb combined with UTMD group than that of the EPI-MBs + mAb without using UTMD group (4.3 ± 1.21% versus 27.2 ± 0.98%, *P* < 0.01), the EPI group (4.3 ± 1.21% versus 16.8 ± 1.15%, *P* < 0.05), or the PBS group (4.3 ± 1.21% versus 32.5 ± 4.54%, *P* < 0.01) (Fig. 2b). Similar efficacy was also found in the mitochondrial membrane potential change (Fig. 2c), which showed a significantly increased mitochondrial membrane potential drop rate in the MM CSCs in the EPI-MBs + mAb combined with UTMD group compared with the EPI-MBs + mAb without UTMD group (35.41 ± 5.53 versus 3.27 ± 1.01%, *P* < 0.01), EPI group (35.41 ± 5.53 versus 13.02 ± 4.80%, *P* < 0.05), or PBS group (35.41 ± 5.53 versus 1.83 ± 0.27%, *P* < 0.01). There were significant differences between the EPI-MBs + mAb combined with UTMD and the EPI-MBs + mAb groups and between the EPI-MBs + mAb combined with UTMD and the EPI groups (Fig. 2d).

Subsequently, we analyzed the effect of different agents on the cell cycle and apoptosis of MM CSCs by FCM. The data presented in Fig. 2e shows that the highest percentage of G1 phase cell count was in the EPI-MBs + mAb combined with UTMD group, which was statistically significant compared with the EPI-MBs + mAb without UTMD group (69.54 versus 33.68%, *P* < 0.01), EPI group (69.54 versus 46.29%, *P* < 0.05), or PBS group (69.54 versus 34.32%, *P* < 0.01). In contrast to the G1 phase, the cell count in the S phase was lower in the EPI-MBs + mAb combined with UTMD group than that in the EPI-MBs + mAb without UTMD group (19.07 versus 60.73%, *P* < 0.01), EPI group (19.07 versus 44.64%, *P* < 0.05), or PBS group (19.07 versus 62.49%, *P* < 0.01) (Fig. 2f). All the data from the in vitro experiments demonstrated that the EPI-MBs + mAb combined with US exposure had a significant efficacy on inhibition of clone formation and induction of MM CSC apoptosis, suggesting the UTMD destroys the MBs and releases EPI from the MBs to the site of MM CSCs, and promotes EPI to easily enter cells through perforations in CSCs.

### Analysis of EPI-MBs + mAb-targeted binding to MM tissues

**Fig. 3:**
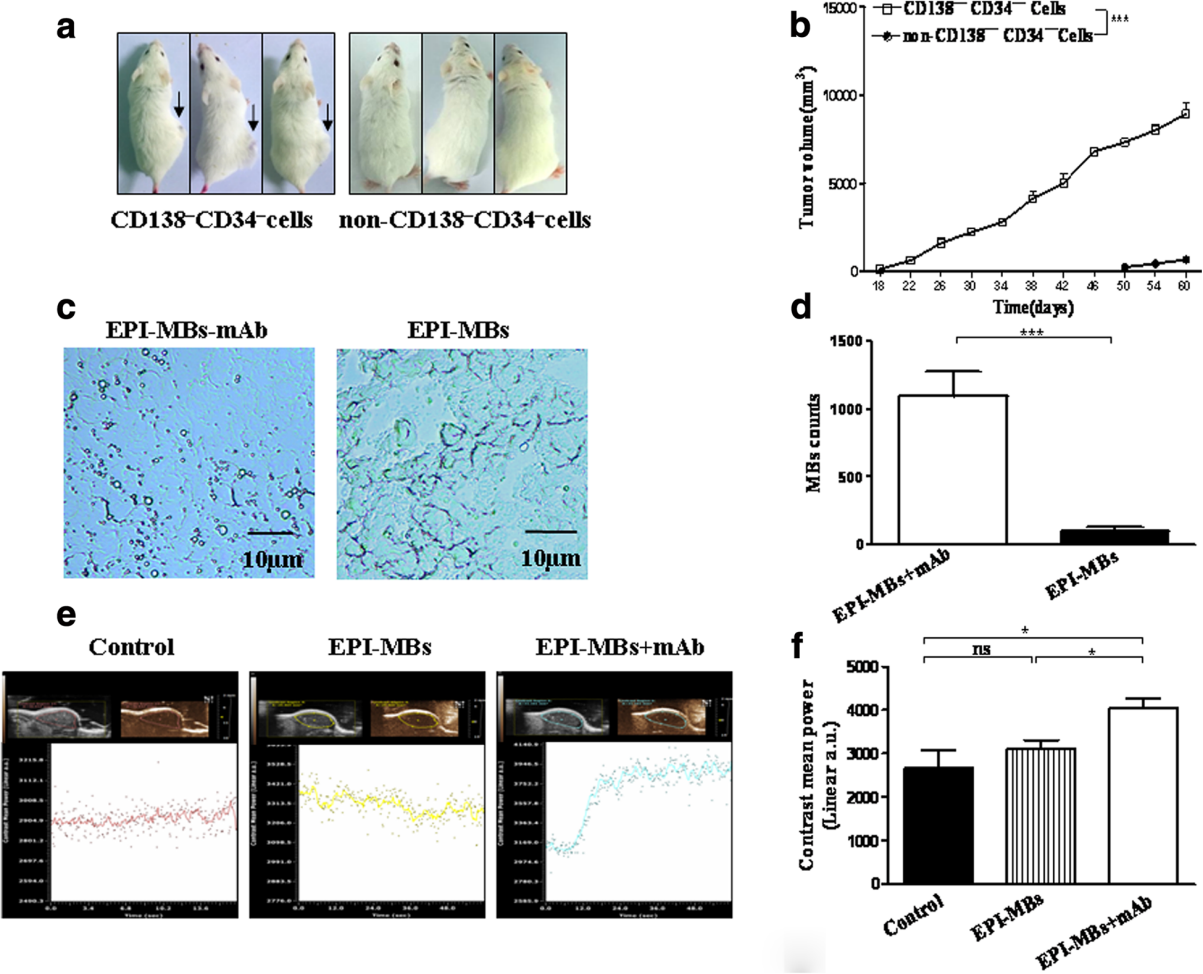
Detection of EPI-loaded MBs in MM tissues. **a** Images of tumor-bearing mice on day 34 after 1 × 10^6^ MM CD138^−^CD34^−^ cells or non-MM CD138^−^CD34^−^ cells were injected s.c. into NOD/SCID mice. **b** Dynamic tumor growth curve. **c** MBs were located in the MM tissues of mice injected with EPI-MBs + mAb (left) or EPI-MBs (right) 30 min after injection. **d** Quantification analysis was performed on tumor sections observed under light microscopy according to MB counts in EPI-MBs + mAb and EPI-MBs groups. **e** Ultraphonic echo intensity was analyzed as described in the Methods. **f** Ultraphonic echo intensity of statistical comparisons referring to G1 or S. *P < 0.05, *** *P* < 0.005. EPI, epirubicin; mAb, monoclonal antibody; MB, microbubble; ns, not significant

To show whether the EPI-MBs + mAb could target binding to MM tissues, we first established the MM-bearing mouse model. Figure 3a shows images of MM-bearing mice (Fig. 3a, left) 34 days after 1 × 10^6^ MM CD138^−^CD34^−^ cells were injected s.c. into the right dorsal side in NOD/SCID mice, but no tumor (Fig. 3a, right) was found in mice injected with 1 × 10^6^ non-MM CD138^−^CD34^−^ cells. Figure 3b depicts the dynamic state curve of tumor growth in mice, suggesting the MM CD138^−^CD34^−^ cells have CSC features such as self-renewal and tumorigenicity.

Next, we injected EPI-MBs + mAb or EPI-MBs into the MM-bearing mice through the tail vein. The images 30 min after injection show that a lot of MBs were located in the MM tissues of mice injected with EPI-MBs + mAb (Fig. 3c); however, only a few MBs were found in mice injected with EPI-MBs, which was statistically significant (*P* < 0.01; Fig. 3d). The results suggested that EPI-MBs + mAb could directly target binding to MM tissues owing to the specific anti-ABCG2 monoclonal antibody.

Further, we evaluated the ultraphonic echo intensity in the tumor tissues by ultrasonic testing. Representative images in Fig. 3e show the stronger ultraphonic echo intensity in the EPI-MBs + mAb group than in the EPI-MBs group or control group, which was statistically significant (*P* < 0.05; Fig. 3f). These data further supported that the ability to bind to MM tissue for EPI-MBs + mAb plus UTMD was better than that of EPI-MBs plus UTMD without mAb in the treatment of MM in vivo*.*

### Evaluation of the effect of EPI-MBs + mAb combined with UTMD on MM-bearing mice

**Fig. 4:**
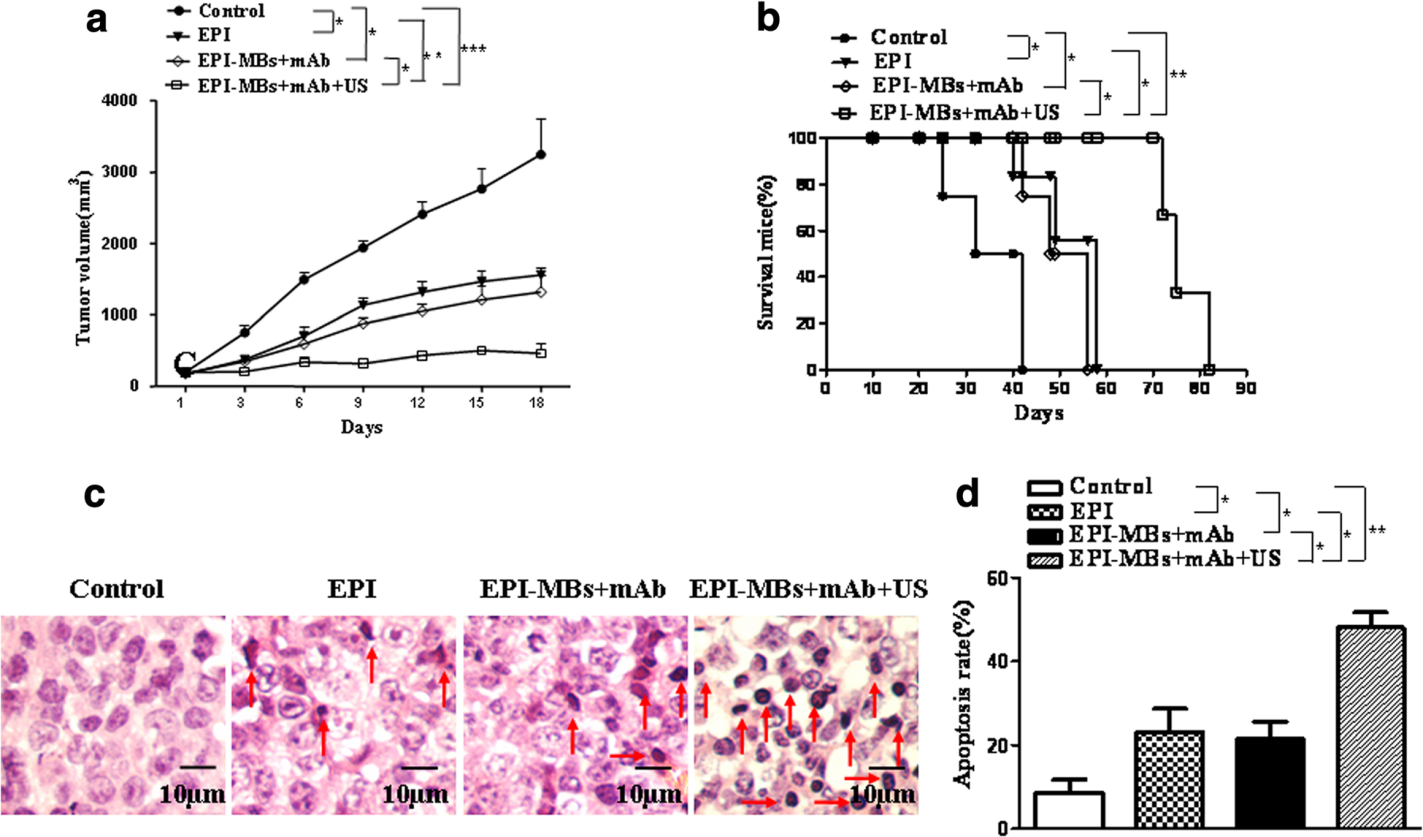
Therapeutic effect of EPI-MBs + mAb combined with ultrasound exposure on MM CD138^−^CD34^−^ CSC-derived tumors in NOD/SCID mice (six per group). **a** Dynamic tumor volumes. The MM-bearing NOD/SCID mice were treated with the different agents as described in the Methods. **b** Quantification analysis of survival time. **c** Tumor histopathologic changes (hematoxylin and eosin; original magnification, ×400) in MM-bearing mice treated with the different agents. **d** Quantification of apoptosis. *P < 0.05, **P < 0.01, ***P < 0.005. EPI, epirubicin; mAb, monoclonal antibody; MB, microbubble; US, ultrasound

Since EPI-MBs + mAb could directly target the local MM tissue in mice, we sought to evaluate whether this targeted binding ability would effectively inhibit tumor growth in the MM CSC xenograft mouse model. The data presented in Fig. 4a show that all treated mice developed smaller tumors than did control mice without treatment but, in the EPI-MBs + mAb combined with UTMD group, the tumor volumes were significant smaller than in the EPI group (*P <* 0.01) or in the EPI-MBs + mAb without US group (*P <* 0.05). In addition, mice treated with EPI-MBs + mAb combined with UTMD showed longer survival time than mice treated with the other agents in this study (Fig. 4b). There was a significant difference between the EPI-MBs + mAb combined with UTMD group and the EPI-MBs + mAb group (*P <* 0.05), between the EPI-MBs + mAb combined with UTMD group and the EPI group (*P <* 0.01), and between the EPI-MBs + mAb combined with UTMD group and the control group (*P <* 0.01).

Tumor histopathologic section images in Fig. 4c show that the tumor cells were distributed sparsely, and that some necrotic or apoptotic tumor cells were more noticeable in tumor tissues of mice treated with EPI-MBs + mAb combined with UTMD than those in mice treated with a single agent or combination agent without UTMD, which was statistically significant (*P <* 0.05 or *P <* 0.01; (Fig. 4d).

### Detection of apoptosis pathway-related molecule expression in tumor tissues

**Fig. 5:**
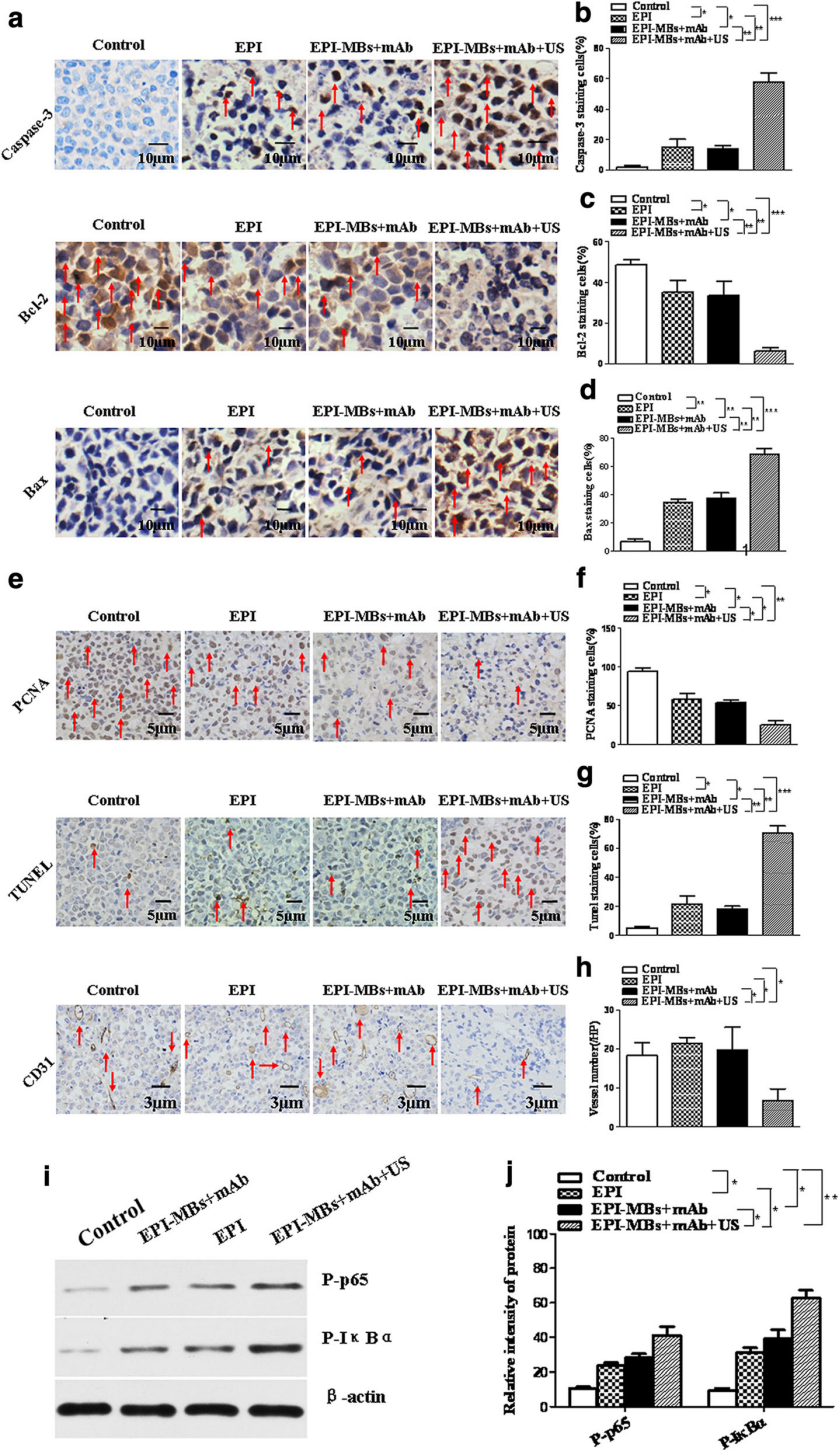
Expression of apoptosis-related molecules in tumor tissues analyzed with immunohistochemistry and Western blot. **a,e** Images of apoptosis-related molecule expression, including Caspase-3, Bax, TUNEL, PCNA, Bcl-2, and CD31 analyzed with immunohistochemistry (**a**, original magnification ×400; **e**, original magnification ×100). **b–d,f–h** Quantification analysis of Caspase-3, Bax, TUNEL, PCNA, Bcl-2, and CD31 expression. **i** Expression of P-p65and P-IκBα analyzed by Western blot. **j** Semiquantification analysis of molecular expression. *P < 0.05, **P < 0.01. EPI, epirubicin; mAb, monoclonal antibody; MB, microbubble; US, ultrasound

To understand the anti-MM mechanisms of EPI-MBs + mAb combined with therapeutic ultrasound, we analyzed the related molecule expression in tumor tissues from mice using immunohistochemistry assay. Figure 5a, e shows the expression of Caspase-3, Bax, TUNEL, PCNA, Bcl-2, and CD31. We found that the tumor cells from the mice treated with EPI-MBs + mAb combined with UTMD significantly increased the staining of Caspase-3, Bax, and TUNEL, and remarkably decreased the staining of PCNA, Bcl-2, and CD31, compared to those in the tumor cells from the mice treated with EPI-MBs + mAb, or EPI, or PBS control (*P <* 0.05 or *P <* 0.01 or *P <* 0.005; Fig. 5b–d, f–h). Meanwhile, the expression of P-p65 and P-IκBα as analyzed by Western blot (Fig. 5i) was also increased in the EPI-MBs + mAb combined with UTMD group, which was statistically significant compared with the EPI-MBs + mAb (*P <* 0.05), EPI (*P <* 0.05), and PBS groups (*P <* 0.01) (Fig. 5j).

## Discussion

MM is a terminally differentiated plasma cell malignancy for which no consistently curative treatment exists. The molecular mechanisms underlying MM chemoresistance remain elusive. One possible explanation is that CSCs appear to be relatively resistant to standard anticancer therapies by co-opting intrinsic defense mechanisms of normal stem cell, such as quiescence, efflux pumps, and detoxifying enzymes [31, 32]. Therefore, there is a continued and unmet need to expand the therapeutic arsenal against this disease [24, 33]. Emerging evidence supports the view that targeting MM CSCs may increase the therapeutic effectiveness against MM [34]. In this regard, we focused on a combination of a conventional drug against MM with targeted ABCG2 ability as well as a UTMD technique to design an exciting therapeutic strategy for the treatment of the refractory disease in MM-bearing NOD/SCID mice.

To assess the effect of the therapeutic strategy on CD138^−^CD34^−^ CSCs isolated from the human MM RPMI8226 cell line, we examined the capability for clonogenic formation and induction of apoptosis of MM CSCs in vitro. From the results of the colony formation assay, we found that the EPI-MBs + mAb combined with UTMD treated MM CSCs showed the lowest colony count among the four groups. It is known that the clone formation assay in the soft agar can be used to measure the ability of cells to cross tissue barriers and to measure the cell invasion, and that the cloning efficiency represents the cellular proliferative and self-renewal ability that is correlated positively with the disease stages of MM, plasma cell leukemia, or advanced MM [1, 24, 35]. Therefore, the data presented in our soft agar clone assay suggested that the EPI-MBs + mAb combined with UTMD could effectively inhibit the proliferation of MM CSCs, and this was further supported by the apoptosis and cell cycle assays. These results indicated that MM CSC apoptosis and cell cycle blocking were significantly higher in the EPI-MBs + mAb combined with UTMD group compared with any other groups.

Furthermore, the in vivo therapeutic results for EPI-MBs + mAb combined with UTMD revealed stronger efficacy for inhibiting MM CSC-derived tumor growth and increasing MM-bearing NOD/SCID mouse survival than that of EPI-MBs + mAb without UTMD or using EPI alone. Obviously, the combination of EPI-loaded MBs with conjugated ABCG2 mAb plus UTMD could target CSCs for ultimately a better treatment of MM. The target-specific CSC inhibition mediated by several indirect mechanisms may be involved in the MBs conjugated with mAb directly binding to the ABCG2 molecule on the surface of MM CSCs, shown in Fig. 3c, which could cause EPI delivery from the MBs to MM CSCs under ultrasound action. In addition, the mAb can partly block the ABCG2 efflux pumps and reserve EPI in MM CSCs for developing a cytotoxic effect on tumor cells as shown in Fig. 4c. This synergetic action resulted in MM growth inhibition.

We hypothesize that the inhibitory activity of EPI-MBs + mAb combined with UTMD may be involved in induction of MM CSC apoptosis. This hypothesis was supported by the data from the immunohistochemistry assay. We found that the characteristic apoptosis molecules, such as Caspase-3, Bax, and TUNEL, were significantly increased in the tumor tissues of mice treated with the combination of EPI-MBs + mAb plus UTMD, whereas the inhibition of apoptosis and proliferation as well as promotion of vascularization molecules (PCNA, Bcl-2, and CD31) were respectively decreased compared with any other groups. Meanwhile, Western blot data showed that the expression of P-p65 and P-IκBα, NF-κB signal pathway-related molecules, was synchronously increased in tumor cells from the mice treated with EPI-MBs + mAb plus UTMD, suggesting that the NF-κB signal pathway was activated. We presume that the EPI-loaded MBs conjugated with mAb were easily accumulated at tumor tissues, with thus more tumor cell uptake of EPI through perforations under ultrasound exposure, which resulted in increased DNA damage in tumor cells. DNA damage might activate the NF-κB signaling pathway [35].

We acknowledge that, in this study, the precise mechanisms underlying the effect of EPI-MBs + mAb plus UTMD remain unclear concerning the relevance of the apoptosis and NF-κB signaling pathways, particularly with respect to their impact on the synergetic therapeutic MM efficacy. Further investigation is therefore warranted.

## Conclusion

In conclusion, our findings reveal definitive evidence that the EPI-loaded MBs conjugated with ABCG2 mAb plus therapeutic ultrasound can target MM CSCs to develop an effective therapy for MM. The findings suggest an important strategy for a potential target ABCG2 molecule on CSCs for induction of MM CSC apoptosis.

## Abbreviations

CSC: Cancer stem cell
EPI: Epirubicin
mAb: Monoclonal antibody
MB: Microbubble
MM: Multiple myeloma
UTMD: Ultrasound-targeted microbubble destruction
